# Isolation and Characterization of *DkPK* Genes Associated with Natural Deastringency in C-PCNA Persimmon

**DOI:** 10.3389/fpls.2016.00156

**Published:** 2016-02-17

**Authors:** Changfei Guan, Wenxing Chen, Rongli Mo, Xiaoyun Du, Qinglin Zhang, Zhengrong Luo

**Affiliations:** ^1^Key Laboratory of Horticultural Plant Biology, Huazhong Agricultural UniversityWuhan, China; ^2^Institute of Horticultural Sciences, Jiangxi Academy of Agricultural SciencesNanchang, China; ^3^Hubei Collaborative Innovation Center for the Characteristic Resources Exploitation of Dabie MountainsHuanggang, China

**Keywords:** persimmon, PAs, *DkPK*, qRT-PCR, transient over-expression, deastringency

## Abstract

Chinese pollination-constant non-astringent (C-PCNA) persimmon (*Diospyros kaki* Thunb.) is considered to be an important germplasm resource for the breeding of PCNA cultivars, though its molecular mechanisms of astringency removal remain to be elucidated. Previously, we showed that the abundance of pyruvate kinase gene transcripts increased rapidly during astringency removal in C-PCNA persimmon fruit. Here, we report the full-length coding sequences of six novel *DkPK* genes from C-PCNA persimmon fruit isolated based on a complementary DNA (cDNA) library and transcriptome data. The expression patterns of these six *DkPK* genes and correlations with the soluble proanthocyanidin (PA) content were analyzed during various fruit development stages in different types of persimmon, with *DkPK1* showing an expression pattern during the last stage in C-PCNA persimmon that was positively correlated with a decrease in soluble PAs. Phylogenetic analysis revealed that *DkPK1* belongs to cytosolic-1 subgroup, and subcellular localization analysis confirmed that *DkPK1* is located in the cytosol. Notably, tissue expression profiling revealed ubiquitous *DkPK1* expression in different persimmon organs, with the highest expression in seeds. Furthermore, transient over-expression of *DkPK1* in persimmon leaves resulted in a significant decrease in the content of soluble PAs but a significant increase in the transcript levels of pyruvate decarboxylase genes (*DkPDC1, -3, -4, -5*), which catalyze the conversion of pyruvate to acetaldehyde. Thus, we propose that an acetaldehyde-based coagulation effect reduces the content of soluble PAs. Taken together, our results suggest that *DkPK1* might be involved in the natural removal of astringency at the last developmental stage in C-PCNA persimmon. This is the first report to identify several novel full-length *DkPK* genes as well as their potential roles in the natural loss of astringency in C-PCNA persimmon.

## Introduction

Persimmon (*Diospyros kaki* Thunb.) is the most economically important species of the genus *Diospyros*, which is believed to have originated in China. Currently, the species is primarily cultivated in East Asia (Yamada, 2004; Yonemori et al., 2008). Persimmon varieties are classified into two groups according to genetic characteristic of natural deastringency: pollination-constant non-astringent (PCNA) persimmons, which exhibit qualitative traits; and non-PCNA types, which exhibit quantitative traits. PCNA types include Chinese PCNA (C-PCNA) and Japanese PCNA (J-PCNA) persimmons, in which the trait of natural astringency removal is controlled by a single locus that is dominant in the former but recessive in the latter. Non-PCNA types include pollination-constant astringent (PCA), pollination-variant non-astringent (PVNA), and pollination-variant astringent (PVA) types (Akagi et al., 2011). Because the C-PCNA trait is dominant over non-PCNA and J-PCNA, C-PCNA-type persimmon constitutes an important germplasm resource for PCNA cultivar breeding.

Proanthocyanidins (PAs), also known as condensed tannins, are colorless phenolic polymers that accumulate in vacuoles in young persimmon fruits (Yakushiji and Nakatsuka, 2007). These polymers are divided into soluble and insoluble tannins based on their solubility in alcohol, and the fruit astringent sensation is largely caused by the accumulation of soluble tannins. During artificial treatments of some non-PCNA persimmon fruits, the generation of acetaldehyde is thought to be directly involved in soluble tannin coagulation, resulting in astringency removal (Matsuo and Itoo, 1982; Taira, 1995). Artificial deastringency, including treatments with CO_2_, ethanol, or warm water, can trigger the synthesis of acetaldehyde in persimmon fruit (Morton, 1987; Taira, 1995; Salvador et al., 2007). Pyruvate decarboxylase gene *(PDC*) and alcohol dehydrogenase gene (*ADH*) are considered key candidates involved in de-astringency of ‘Mopanshi’ (non-PCNA) persimmon (Min et al., 2012). PDC catalyzes the conversion of pyruvate to acetaldehyde, and ADH is involved in the reversible reaction between acetaldehyde and ethanol (Pesis, 2005; Strommer, 2011). Ethanol or CO_2_ treatment increases the expression of *PDC* and *ADH*, potentially accelerating acetaldehyde synthesis in persimmon fruit (Min et al., 2012; Luo et al., 2014). As opposed to some non-PCNA persimmon, the PCNA type fruits are able to lose astringency naturally on the tree. Several studies have suggested that the natural loss of astringency property in C-PCNA persimmon is significantly different from that in J-PCNA types (Yonemori et al., 2003; Ikegami et al., 2004, 2006). In J-PCNA persimmons, the development of tannin cells stops during the early stages of fruit growth, with astringency removal predominantly occurring via tannin dilution as the fruit grows larger (Yonemori et al., 2003); in addition to the dilution effect that also occurs in C-PCNA types, astringency removal in C-PCNA persimmon is most likely related to soluble tannin coagulation (Zhang et al., 2012). It has been demonstrated that DkMyb4 acts as a key regulator of PA biosynthesis in J-PCNA persimmon, as a reduction in *DkMyb4* expression during the early stages of fruit growth caused a substantial down-regulation of PA pathway genes, including *DkPAL*, *DkCHS*, *DkCHI*, *DkF3H*, *DkF3′5′H*, *DkDFR*, and *DkANR*, and resulting the non-astringent trait (Akagi et al., 2009). However, compared with J-PCNA persimmon, the mechanisms of astringency removal in C-PCNA persimmon fruit are not well understood. Although our recent study revealed significant enrichment of differentially expressed genes related to glycolysis/gluconeogenesis during astringency removal in ‘Luotian-tianshi’ persimmon (Luo et al., 2014), correlations with the molecular mechanisms of astringency removal in C-PCNA persimmon remain to be explored.

Pyruvate kinase (PK; EC 2.7.1.40) is a key regulatory enzyme of the glycolytic pathway that catalyzes the irreversible synthesis of pyruvate and ATP from PEP and ADP. There are abundant data to support that the reaction is a primary control site of plant glycolytic flux toward pyruvate (Plaxton, 1996). As opposed to non-plant PKs, both cytosolic (PKc) and plastid (PKp) isozymes exist in plants, which differ markedly in their respective physical, immunological and kinetic/regulatory characteristics (Plaxton, 1996; Plaxton et al., 2002). PKp is thought to be critical for supplying pyruvate and ATP to several plastidic biosynthetic pathways (Knowles et al., 1998; Andre et al., 2007; Oliver et al., 2008), whereas PKc function is more complicated because it plays a major role in a range of biosynthetic pathways as well as respiration (Knowles et al., 1998; Oliver et al., 2008). For example, PKc was reported to respond to several defense-related abiotic stresses in *Capsicum annuum* (Kim et al., 2006) and to regulate pyruvate and alternative oxidase levels in heterotrophic tissues in potato (Oliver et al., 2008). PKc also plays a potential regulatory role in fruit ripening (Law and Plaxton, 1997; Qin et al., 2013), with a stronger PKc mRNA expression resulting in a higher fruit sugar content in loquat fruit (Qin et al., 2013). In our previous work, we found that PKc expression was notably increased during astringency removal in C-PCNA persimmon fruits treated with warm water (Guan et al., 2015). Nonetheless, the role of PKc in natural deastringency in C-PCNA persimmon fruit has not yet been investigated.

In the present research, full-length complementary DNAs (cDNAs) of *DkPK* genes (Accession Nos. KU130358 to KU130363) were isolated from C-PCNA persimmon fruit based on a cDNA-sequence specific amplification polymorphism (SSAP) library and transcriptome data (Luo et al., 2014; Guan et al., 2015). Next, the transcript abundance of *DkPK* genes and correlations with soluble PAs were analyzed at different developmental stages of C-PCNA, J-PCNA, and non-PCNA persimmon fruits. Furthermore, transient over-expression was used to confirm the function of candidate *DkPK* genes in persimmon leaves. Our study will be helpful for understanding the mechanism of astringency removal and for the breeding of PCNA cultivars in the future.

## Materials and Methods

### Materials

Three representative persimmon (*D*. *kaki* Thunb.; 2n = 6x = 90) cultivars, ‘Luotian-tianshi’ (C-PCNA) as the main material and ‘Youhou’ (J-PCNA) and ‘Mopanshi’ (non-PCNA) as controls, were sampled from the Persimmon Repository, Huazhong Agricultural University, Wuhan, China. Fruit flesh samples from uniform fruits free of visible defects were collected at 2.5, 5, 10, 15, 20, and 25 weeks after flowering (WAF), at different developmental stages. Leaf, stem, sepal, peel, pulp, seed, and core samples were collected for tissue-specific gene expression at 22 WAF, and flowers were harvested at full bloom. Twelve fruits or other tissues were sampled for each treatment, and each treatment was repeated three times. All the samples were frozen in liquid nitrogen and stored at -80°C.

### PA Content Analysis

Insoluble and soluble PAs were measured by the Folin–Ciocalteu method (Oshida et al., 1996), with absorbance measured at 725 nm using a UV-2450 spectrophotometer (Shimadzu, Japan).

### RNA Extraction and cDNA Synthesis

Total RNA was isolated from frozen flesh samples using RNAplant Plus Reagent (Tiangen Biotech Co., Beijing, China). The RNA quality and quantity were assessed by gel electrophoresis and Nanodrop 2000 spectrophotometry (Thermo Fisher, Waltham, MA, USA). Three biological replicates were performed for each sample. For gene isolation, first-strand cDNA was generated using 2 μg total sample RNA with EasyScript One-Step gDNA Removal and cDNA Synthesis SuperMix Kit (TransGen, Beijing, China) according to the manufacturer’s protocol. For gene expression analysis, cDNA was synthesized from 1.0 μg of each RNA sample using PrimeScript RT Kit with gDNA Eraser (TaKaRa, Dalian, China).

### Gene Isolation and Sequence Analysis

pyruvate kinase genes were isolated based on a cDNA-SSAP library and RNA-Seq data for C-PCNA persimmon. Both data sets have been previously reported by our group (Luo et al., 2014; Guan et al., 2015). Full-length cDNAs of *PK* genes were amplified with a SMART rapid amplification of cDNA ends (RACE) cDNA Amplification Kit and Universal GenomeWalker Kit (Clontech, USA). The sequences of the primers used for RACE, genome walking and cloning are described in Supplementary Table S1. The gene sequences were translated with online software (http://linux1.softberry.com/) and confirmed with BLAST methods in GenBank (Benson et al., 2000). Deduced amino acid sequences of homologous genes in other species were retrieved from National Center for Biotechnology Information (NCBI) and Oliver et al. (2008). A phylogenetic tree was constructed using the neighbor-joining (NJ) method with the MEGA6 software (Tamura et al., 2011).

### Quantitative Reverse Transcription PCR (qRT-PCR)

Quantitative reverse transcription PCR was performed with a LightCycler^®^480 II System (Roche Diagnostics, Rotkreuz, Switzerland). The PCR reaction mixture (20 μl total volume) included 10 μl SYBR Premix Ex Taq II (TaKaRa, Dalian, China), and 7.4 μl ddH_2_O, 1.0 μl diluted cDNA, and 0.8 μl each primer (10 μM). The PCR conditions were as follows: 5 min at 95 °C, 45 cycles of 95°C for 5 s, 58°C for 10 s, and 72°C for 15 s, and a melting temperature cycle with constant fluorescence data acquisition from 65 to 95°C. Each sample was assayed in quadruplicate, and *DkActin* (Akagi et al., 2009) was used as the internal control (Supplementary Table S1).

### Subcellular Localization in *Nicotiana benthamiana*

To determine the subcellular localization of DkPK1, the complete open reading frame (ORF) of *DkPK1* without the termination codon was amplified using the forward primer 5′-CGGGATCCATGCACGCGAATCATCTTCT-3′ and the reverse primer 5′-GGGGTACCAGGCCATAGTTCCCAACACTC-3′. The PCR amplification products and the 101LYFP binary vector were both digested with *Bam*H I/*Kpn* I, and the ORF sequence was ligated to the vector to generate the fusion construct 35S-DkPK1::YFP; 35S-YFP was used as a control. The fusion and control plasmids were transferred into *Agrobacterium tumefaciens* strain GV3101 by heat shock and then transiently transformed into leaves of 6-week-old *N. benthamiana* plants. Two days later, the fluorescence signal was analyzed under a confocal laser scanning microscopy (CLSM, Olympus Fluoview FV1000, Japan).

### Transient Transformation of Persimmon Leaves *In Vivo*

A transient over-expression system was utilized to assess the role of *DkPK* genes in the coagulation of soluble tannins in persimmon leaves *in vivo*. The full-length coding region of *DkPK1* was amplified by reverse transcription PCR (RT-PCR) with the following primers: forward, 5′-GCTCTAGAGGTGCGAATCTTCAGAGTCCT-3′, and reverse, 5′-GGGGTACCAGGCCATAGTTCCCAACACTC-3′. The amplified product was cloned into binary vector pMV2 after digesting with *Xba* I/*Kpn* I. The construct was transferred into ‘Mopanshi’ persimmon leaves via a previously described *Agrobacterium*-mediated method, with minor modifications (Mo et al., 2015). The *Agrobacterium* cells collected by centrifugation were re-suspended to an optical density (OD) at 600 nm of 0.60. The pMV2-GFP vector was used as an empty vector control. The transformed leaf still grew on the tree before they were isolated for qRT-PCR and tannin content analysis. Eight days later, 100 mg tissue from each infiltrated leaf was collected for further experiments, with a total of ten single leaf replicates.

## Results

### Changes of PA Content During Fruit Development in Different Types of Persimmon

**FIGURE 1:**
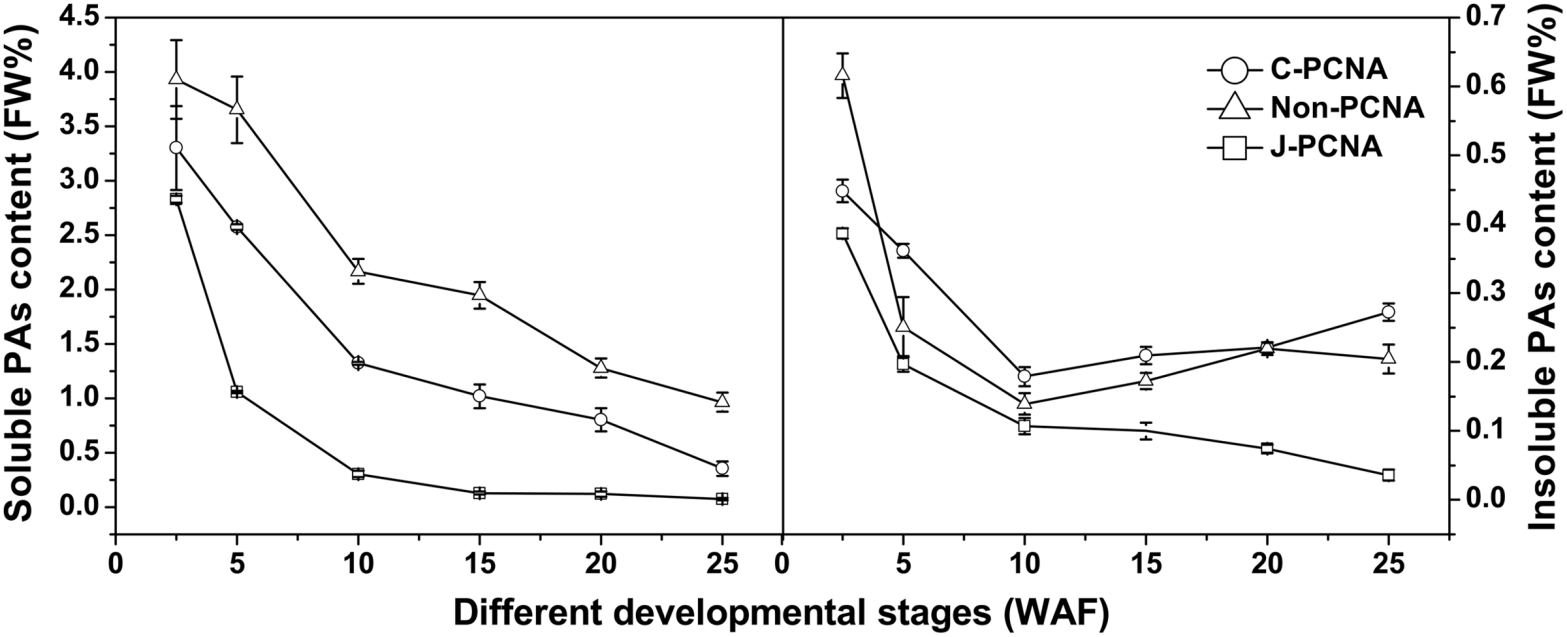
**Changes in proanthocyanidin (PAs) during fruit development in different types of persimmon.** Soluble and insoluble tannins were measured according to the Folin-Ciocalteu method. ‘Luotian-tianshi’ is a C-PCNA-type persimmon, ‘Youhou’ a J-PCNA type and ‘Mopanshi’ a non-PCNA type. Flesh was collected at 2.5, 5, 10, 15, 20, and 25 WAF. FW%, the tannin concentration per fresh weight; WAF, weeks after flowering.

Obvious differences in soluble PA content were observed among C-PCNA, non-PCNA and J-PCNA persimmons. During the entire fruit development process, the soluble PA content of C-PCNA was between that of non-PCNA and J-PCNA (**Figure 1**). However, significant decreases in soluble PA content among the three types were noted during fruit development, indicating a dilution effect of soluble PAs in different types of persimmon as the fruits grow larger.

For the J-PCNA cultivar, astringency was removed at 15 WAF, and a low level of soluble PA content was maintained until the last stage. Among the three cultivars, insoluble PAs in the J-PCNA type exhibited the lowest concentration and decreasing trend during fruit development, consistent with the previous result that the development of tannin cells stops during the early stages of J-PCNA persimmon fruit growth (Akagi et al., 2009). In contrast, the non-PCNA cultivar showed a high PA content until the last developmental stage (20–25 WAF), though without natural astringency removal (**Figure 1**). Compared with the non-PCNA type, the soluble PA content of C-PCNA persimmon fruits was significantly decreased from 0.8% at 20 WAF to the edible level (nearly 0.2%) at 25 WAF. These results indicate that the coagulation of soluble tannins in the last developmental stage would most likely account for the natural loss of astringency in C-PCNA fruit.

### Gene Isolation and Sequence Analysis of *DkPK1-6*

**FIGURE 2:**
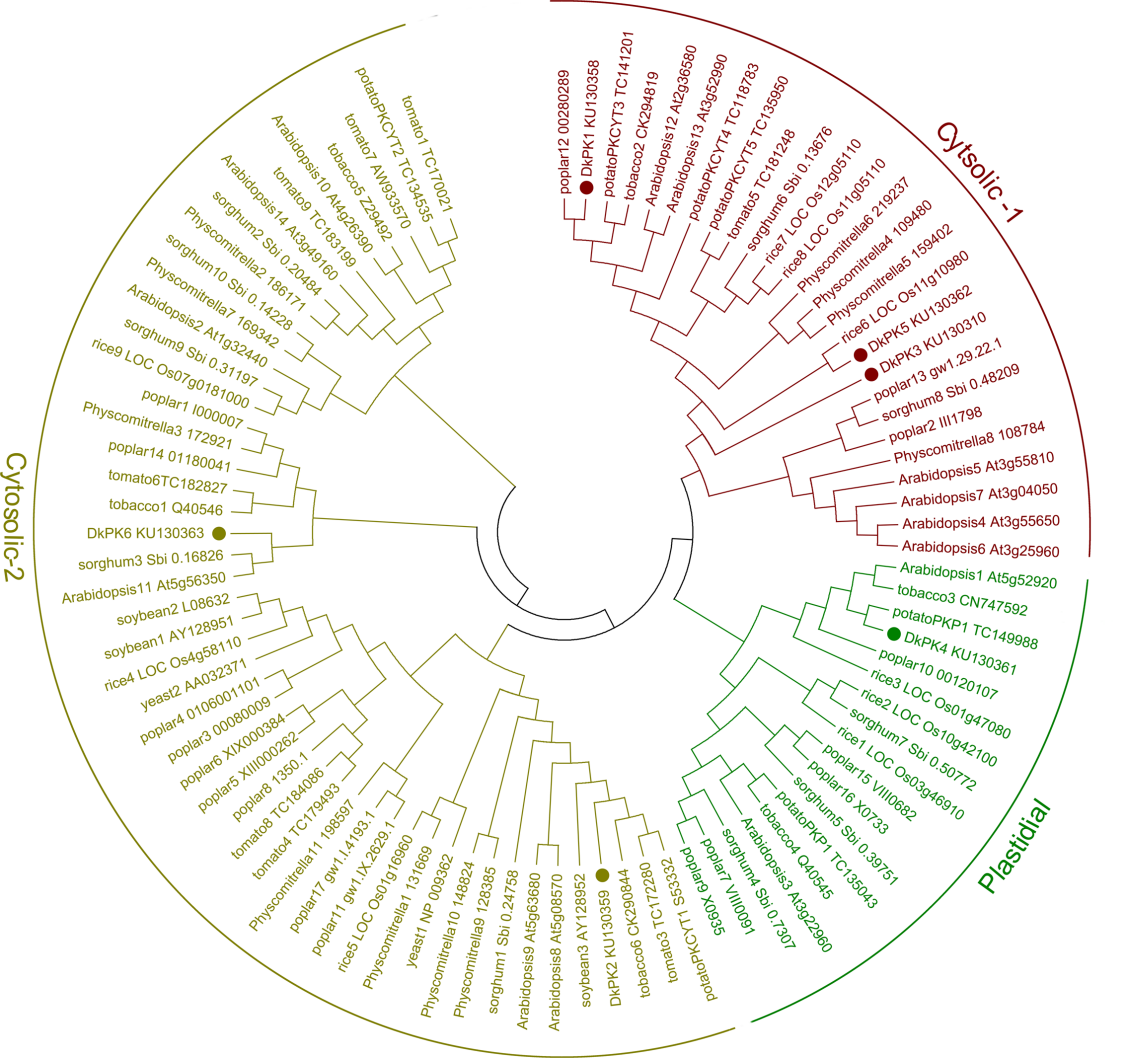
**Phylogenetic tree of pyruvate kinase (PK) proteins.** The deduced amino acid sequences of PKs were obtained from NBCI and (Oliver et al., 2008); the accession numbers are indicated. Persimmon PK proteins are shown in bold. The phylogenetic tree was constructed using MEGA6.

The full-length cDNAs of six *DkPK* genes (*DkPK1*-*6*) isolated from C-PCNA persimmon fruit were obtained by cDNA-SSAP and RNA-Seq data. Phylogenetic analysis of the deduced protein sequences showed clear separation of the plastidial and cytosolic PK isozymes, and cytosolic PK was further divided into two subgroups: cytosolic-1 and cytosolic-2 (**Figure 2**). In the phylogenetic tree, DkPK1, DkPK3, and DkPK5 were clustered together in cytosolic-1 subgroup, DkPK2 and DkPK6 grouped with the cytosolic-2 subfamily, and DkPK4 were classified into the plastidial group. The putative cellular localization of DkPK is also supported by BLAST and the prediction program WoLF PSORT (Horton et al., 2007).

### Expression Patterns of *DkPK1-6* During Fruit Development in Different Types of Persimmon

**FIGURE 3:**
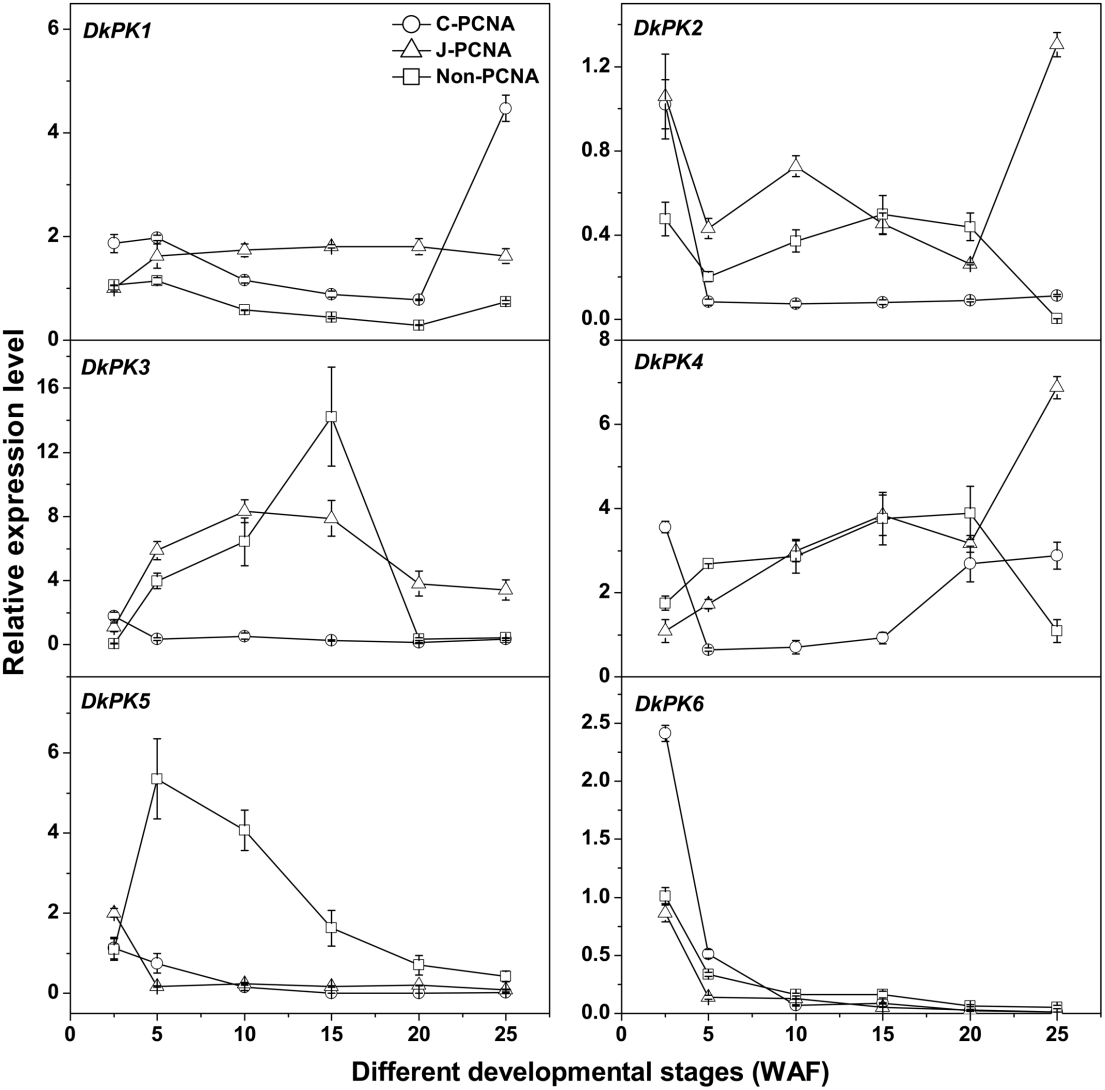
**Expression analysis of *DkPK1-6* during fruit development in different types of persimmon. ‘Luotian-tianshi’ is a C-PCNA-type persimmon, ‘Youhou’ a J-PCNA type and ‘Mopanshi’ a non-PCNA type.** Flesh was collected at 2.5, 5, 10, 15, 20, and 25 WAF. WAF, weeks after flowering. *Error bars* indicate the standard deviation (*n* = 3).

To better understand the correlations between the patterns of *PK* gene expression and changes in the PA content in C-PCNA persimmon, the six *DkPK* genes were further analyzed using qRT-PCR at different C-PCNA persimmon developmental stages (Supplementary Table S1 and **Figure 3**). J-PCNA and non-PCNA cultivars were used as control. One of the most interesting results was that *DkPK1* expression in C-PCNA persimmon increased by sixfold during the last stage (20–25 WAF), displaying a negative correlation with the content of soluble PAs at the same stage. Conversely, the expression of *DkPK1* was relatively constant in both J-PCNA and non-PCNA persimmon fruit throughout development. The transcript abundance of *DkPK2* and -*3* and *DkPK5* in C-PCNA persimmon decreased rapidly at 5 and 10 WAF, respectively, though the expression level of each was relatively constant until the last stage. In both C-PCNA and J-PCNA, the level of *DkPK4* expression peaked at 25 WAF, and *DkPK6* exhibited a similar expression pattern in C-PCNA, J-PCNA, and non-PCNA persimmon fruits, presenting a decreasing tendency throughout development. Combining the *DkPK1* expression pattern, which exhibited a negative correlation with the soluble PA content, we propose that *DkPK1* is most likely to be involved in natural deastringency in C-PCNA persimmon (decrease in soluble PAs or soluble PA coagulation). Hence, we chose *DkPK*1 for further analysis in the present study.

### *DkPK1* was Highly Expressed in the Seeds of C-PCNA Persimmon

**FIGURE 4:**
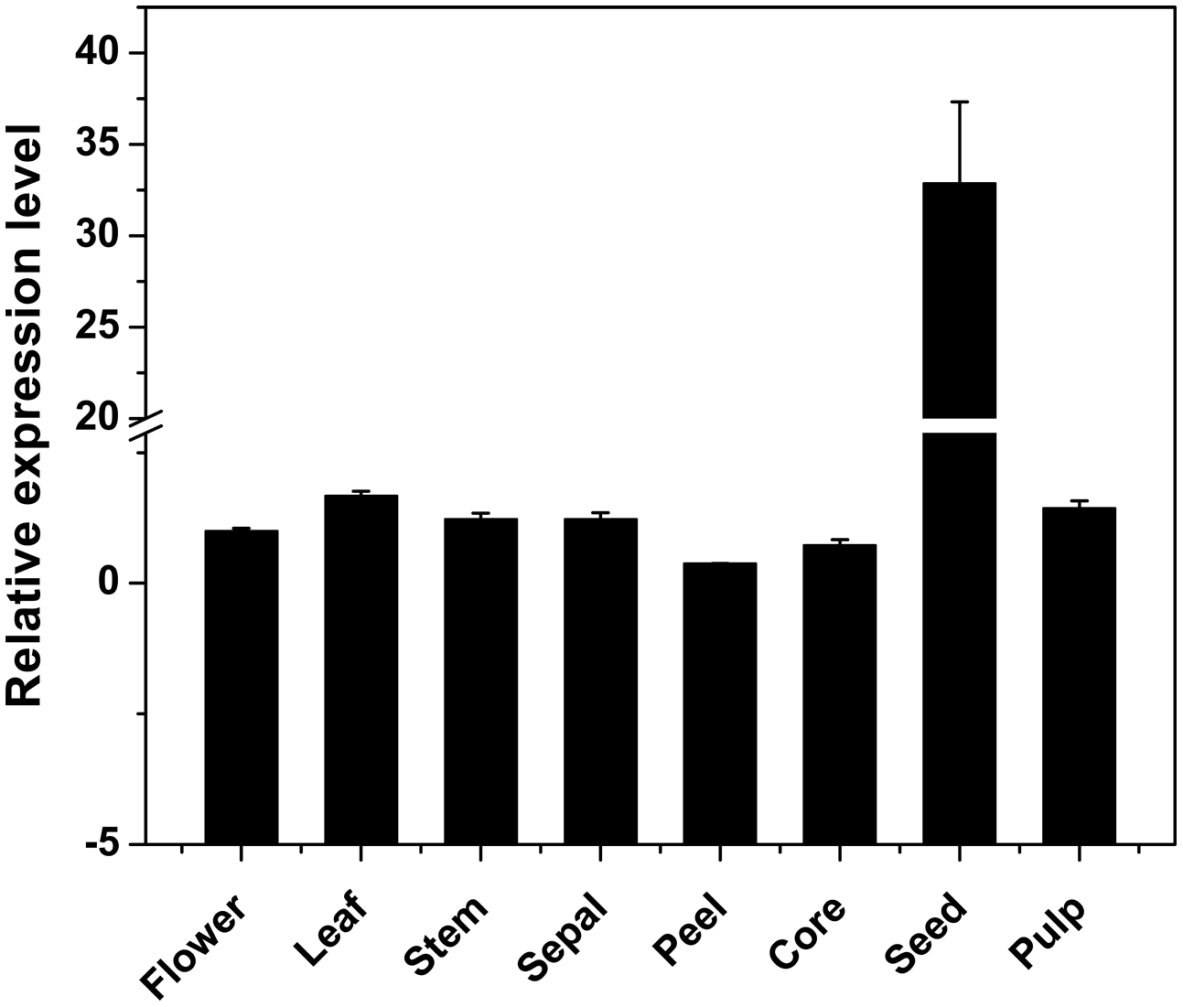
**Relative expression of *DKRE1* in different tissues of ‘Eshi 1’ persimmon.** Leaf, stem, sepal, peel, core, seed, and pulp samples were collected at 22 WAF; flower tissue was collected at the full-bloom stage. *Error bars* indicate the standard deviation (*n* = 3).

Using qRT-PCR, the *DkPK1* transcript patterns were assessed in different tissues, flower, leaf, stem, sepal, peel, core, seed, and pulp, of C-PCNA persimmon. *DkPK1* was found to be ubiquitously expressed in the different persimmon organs, with the highest expression in the seed (nearly 30 times higher than that in the other organs) and the lowest expression in the peel (**Figure 4**). Thus, in view of a previous report that the seeds can release a large amount of volatile compounds, including the acetaldehyde that is involved in the coagulation of soluble tannins in some non-PCNA types (Sugiura and Tomana, 1983), we hypothesize that the high expression of *DkPK1* in seeds might contribute to the acceleration of natural deastringency in C-PCNA persimmon.

### Subcellular Localization of DkPK1

**FIGURE 5:**
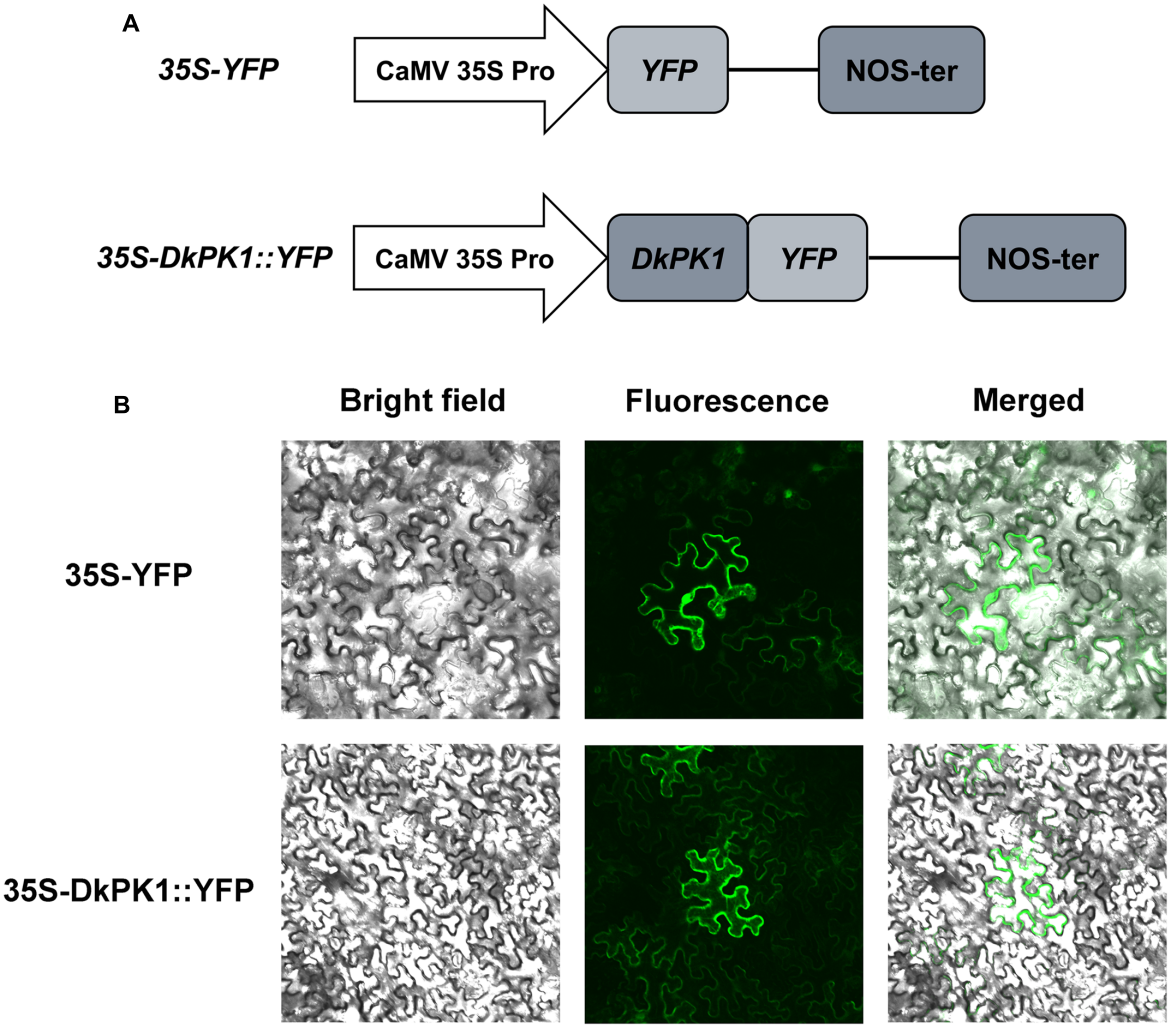
**Subcellular localization of the DkPK1 fusion protein in tobacco leaf epidermal cells.** **(A)** Schematic diagram of the 35S-DkPK1::YFP fusion construct and the *35S-YFP* construct. **(B)** Transient expression of the 35S-DkPK1::YFP and 35S-YFP constructs in tobacco leaf epidermal cells.

As phylogenetic analysis predicted that DkPK1 is located in the cytoplasm, the fusion construct 35S-DkPK1::YFP and the positive control 35S-YFP vector were transiently transformed into tobacco leaf cells via agroinfiltration to further confirm this prediction. The transient expression of 35S-YFP was observed in the cytoplasm and nucleus, whereas YFP fluorescence for the DkPK1 protein was only detected in the cytoplasm, demonstrating that DkPK1 should be regarded as a cytosolic protein (**Figure 5**).

### Over-Expression of *DkPK1* Reduced the Soluble PA Content in Persimmon Leaves *In Vivo*

**FIGURE 6:**
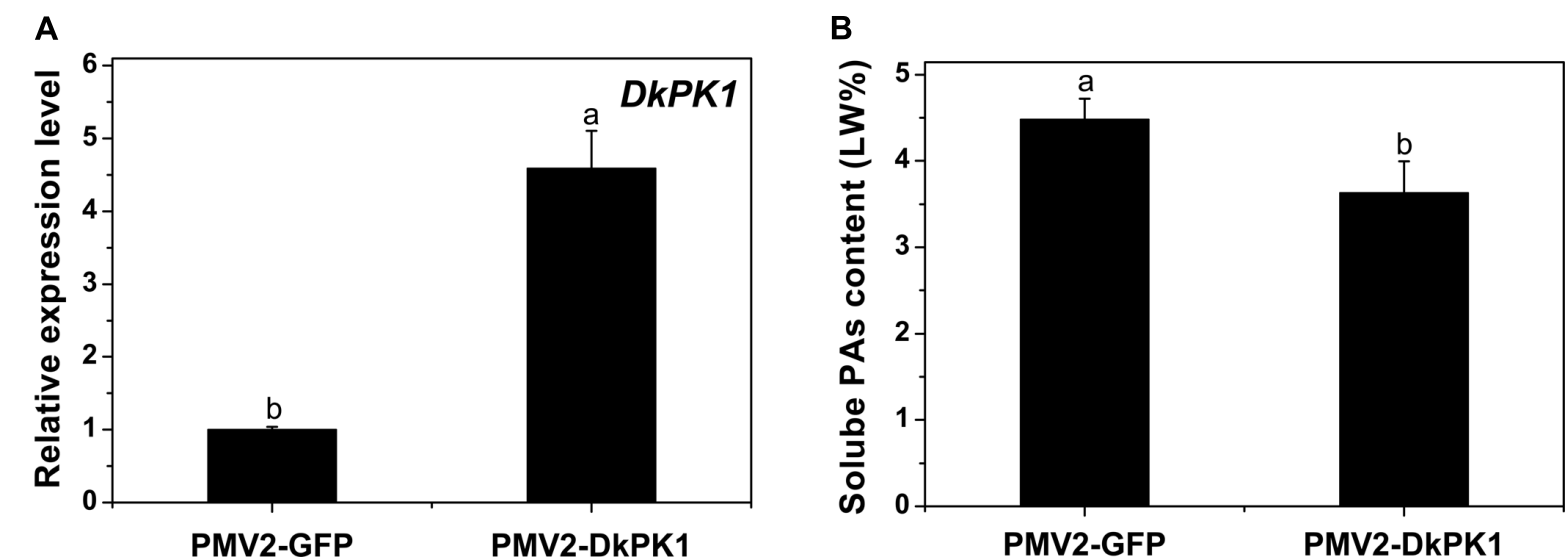
**Transient over-expression of *DkPK1* in ‘Mopanshi’ persimmon leaves.** **(A)** Transcriptional analysis of *DkPK1* and **(B)** changes in soluble PA content after agroinfiltration. Leaves infiltrated with *DkPK1* were collected at 8 days after agroinfiltration. pMV2-GFP represents the empty vector. *Error bars* indicate the standard deviation (*n* = 10). Statistical significance was assessed using one-way analysis of variance (ANOVA) followed by Duncan’s multiple range test (*p <* 0.05).

To confirm the putative function of *DkPK1* in the coagulation of soluble tannins *in vivo*, a transient transformation system in persimmon leaves was chosen for rapid gene functional analysis. The full-length coding sequences (CDS) of *DkPK1* was cloned into the 101LYFP binary vector and then transformed into persimmon leaves via *Agrobacterium*-mediated infiltration. Over-expression of *DkPK1* in ‘Mopanshi’ persimmon leaves was confirmed by qRT-PCR. The level of *DkPK1* transcript in the infiltrated leaves was increased by the 4.5-fold compared with the control (**Figure 6A**). As expected, the level of soluble PAs was significantly decreased in the persimmon leaves infiltrated with *DkPK1* (**Figure 6B**). These results demonstrate that over-expression of *DkPK1* could lead to a significant decrease in the content of soluble tannins in persimmon leaves.

### Over-Expression of *DkPK1* Enhanced the *DkPDC* Transcript Level in Persimmon Leaves *In Vivo*

**FIGURE 7:**
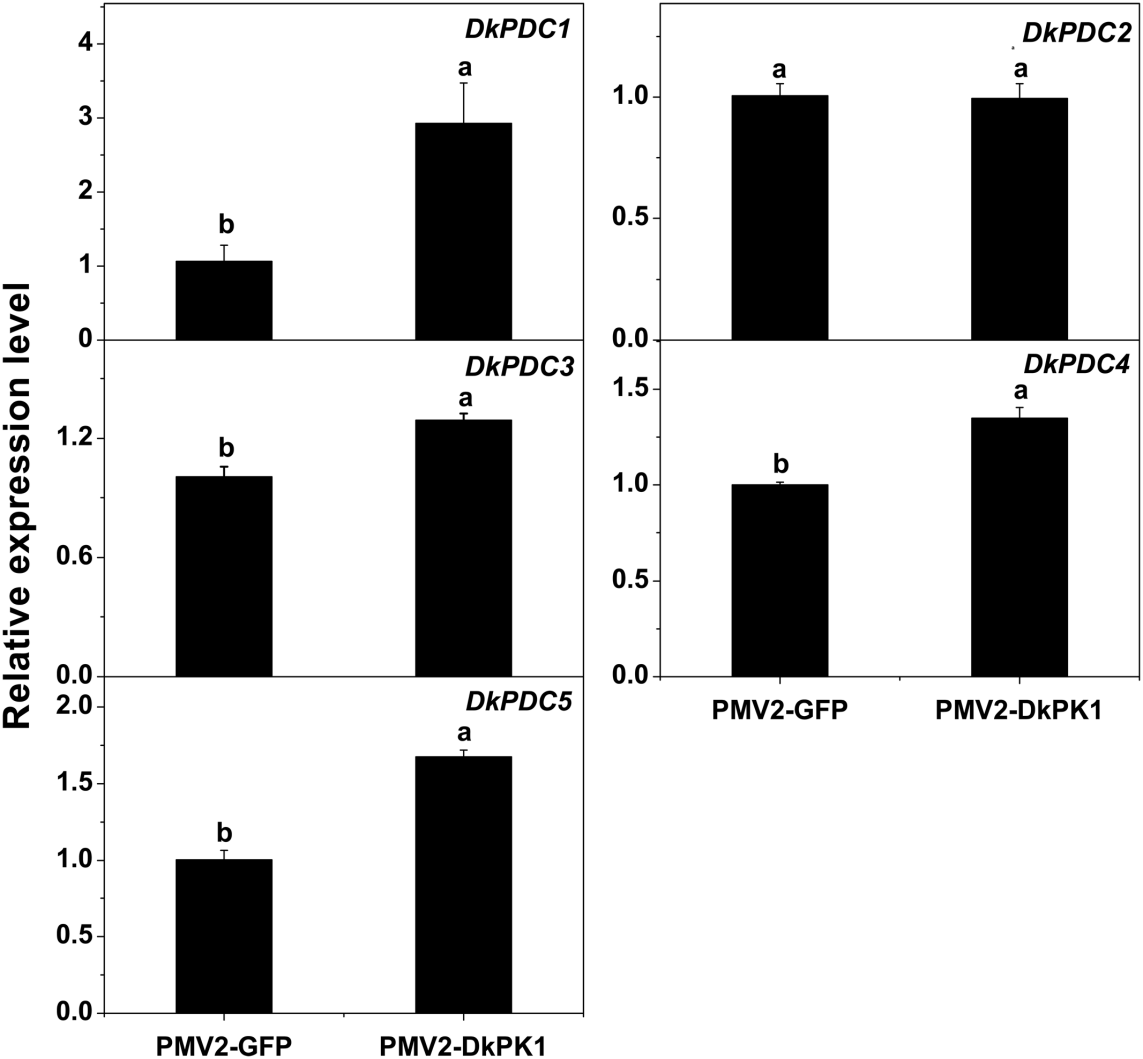
**Over-expression of *DkPK1* enhanced the level of *DkPDC* transcript in persimmon leaves.** Leaves infiltrated with *DkPK1* were collected at 8 days after agroinfiltration. pMV2-GFP represents the empty vector. *Error bars* indicate the standard deviation (*n* = 10). Statistical significance was assessed using one-way analysis of variance (ANOVA) followed by Duncan’s multiple range test (*p <* 0.05).

Pyruvate decarboxylase is thought to be a key enzyme in pyruvate metabolism, catalyzing the conversion of pyruvate to acetaldehyde. Thus, the transcript levels of previously characterized *DkPDC* genes (Min et al., 2012) were also assessed by qRT-PCR in our transiently over-expressing leaves (**Figure 7**). Except for *DkPDC2*, which exhibited fairly constant expression, the expression of *DkPDC1*, *DkPDC3*, *DkPDC4*, and *DkPDC5* was significantly increased in the persimmon leaves infiltrated with *DkPK1.* Moreover, among the four genes, *DkPDC1* was expressed at the highest level, increased by threefold. This result suggests that the transient over-expression of *DkPK1* in persimmon leaves can up-regulate *DkPDCs*, which potentially facilitate the generation of acetaldehyde.

## Discussion

Pyruvate kinase, which is broadly involved in glycolysis and acetaldehyde metabolism, is an important regulatory enzyme for plant growth (Ambasht and Kayastha, 2002). Previous studies have reported that PKc plays a potential regulatory role in loquat and banana fruit ripening (Law and Plaxton, 1997; Qin et al., 2013), and we recently reported significant enrichment of differentially expressed glycolysis/gluconeogenesis-related genes during deastringency in ‘Luotian-tianshi’ (C-PCNA) persimmon (Luo et al., 2014). Furthermore, *DkPK* genes are remarkably up-regulated during astringency removal in ‘Eshi 1’ (C-PCNA) persimmon (Guan et al., 2015). Therefore, we propose that *DkPK* genes might be associated with natural deastringency in C-PCNA cultivars.

In the present study, six *DkPK* genes, designated *DkPK1-6*, were isolated based on cDNA-SSAP and RNA-Seq data using RACE-PCR and genome walking approaches. Next, the expression patterns of six *DkPK* genes and their correlation with the soluble PA content were analyzed during fruit development in different types of persimmon. Among the six genes, the expression pattern of *DkPK1* during the last C-PCNA persimmon development stage was positively correlated with a decrease in the soluble PA content; the expression of *DkPK1* rapidly increased and soluble PA accumulation significantly decreased in last stage (**Figures 1** and **3**). These results indicate that *DkPK1* may be involved in the natural removal of astringency during the last stage of fruit development in C-PCNA persimmon.

**FIGURE 8:**
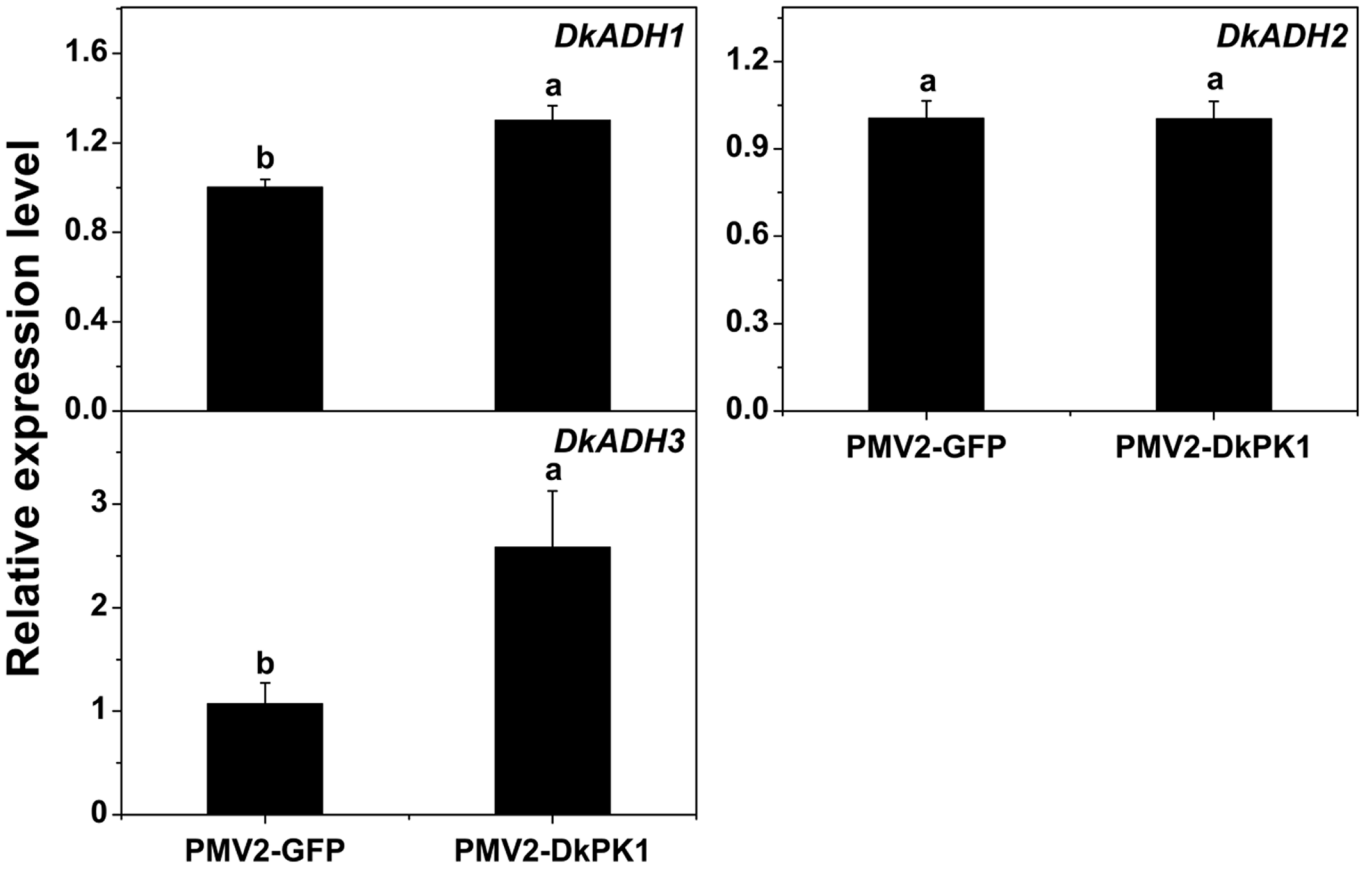
**Over-expression of *DkPK1* enhanced the level of *DkADH* transcript in persimmon leaves.** Leaves infiltrated with *DkPK1* were collected at 8 days after agroinfiltration. pMV2-GFP represents the empty vector. *Error bars* indicate the standard deviation (*n* = 10). Statistical significance was assessed using one-way analysis of variance (ANOVA) followed by Duncan’s multiple range test (*p <* 0.05).

Persimmon is a perennial fruit, and thus a transient transformation system in persimmon leaves was chosen for further rapid gene functional analysis *in vivo*. Transient over-expression of *DkPK1* in persimmon leaves led to a significant reduction in the levels of soluble PAs (**Figure 6**), findings that were in remarkable agreement with the results of expression assays for both *PDC* and *ADH* genes, which are thought to be correlated with astringency removal in persimmon (Min et al., 2012). In the present study, significant increases in the expression of *DkPDC1, -3, -4, -5* as well as *DkADH1* and *-3* were found in leaves infiltrated with *DkPK1* (**Figures 7** and **8**). The relatively constant expression of *DkPDC2* and *DkADH2* in the over-expressing leaves could be attributed to the complex genetic traits of hexaploid persimmon. Considering that up-regulated expression of *DkPDC* and *DkADH* during the process of artificial deastringency is often accompanied by the accumulation of acetaldehyde (Min et al., 2012; Luo et al., 2014), our results suggest that over-expression of *DkPK1* in persimmon leaves might enhance the transcript levels of both *DkPDC* and *DkADH*, which promote acetaldehyde synthesis, resulting in a decrease in soluble PAs.

**FIGURE 9:**
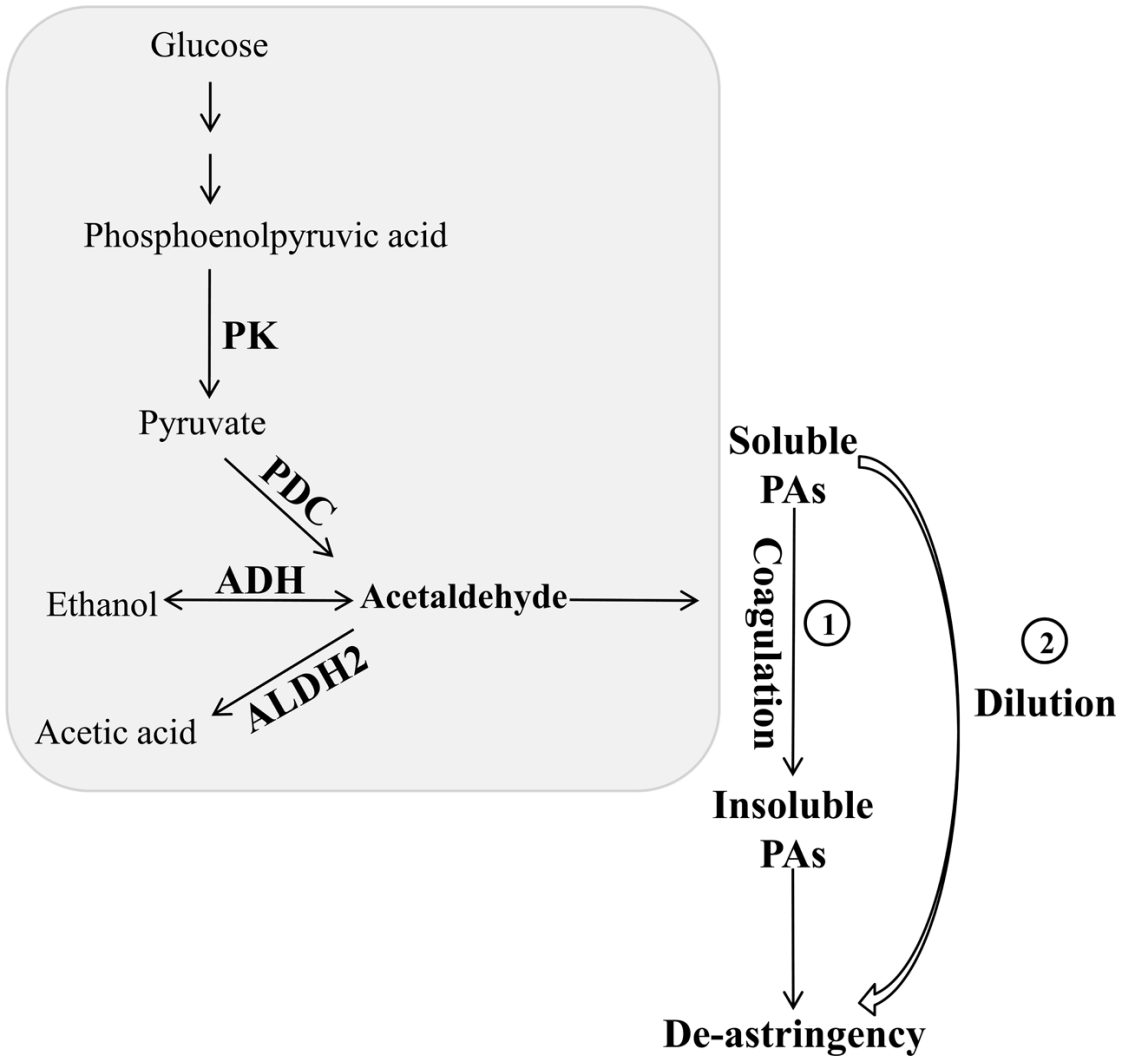
**A hypothetical model of natural deastringency in C-PCNA persimmon.** The acetaldehyde-mediated coagulation effect and the dilution effect as the fruit grows larger may contribute to the natural removal of astringency in C-PCNA cultivars.

Zhang et al. (2012) revealed that natural deastringency in C-PCNA persimmon is not only due to the dilution of tannins as the fruit grows larger but is more importantly, caused by the coagulation of soluble PAs at the last developmental stage. Our results indicate that in addition to the common dilution effect in different types of persimmon, the soluble PA content decreases significantly at the last developmental stage in C-PCNA persimmon (**Figure 1**) while the level of *DkPK1* transcript rapidly increases at the last stage (**Figure 3**). Additionally, the expression of *DkPK1* in the seed was markedly up-regulated, dozens of times higher than in others organs (**Figure 4**). Therefore, taking into account the suggestion of Sugiura and Tomana (1983), that is, the seeds of persimmon release a large amount of volatile compounds involved in the coagulation of soluble tannins in the flesh, we presume that in addition to the expression of *DkPK1* in the flesh, the high expression in seeds could at least partly reduce the soluble PA level in C-PCNA persimmon fruits. Finally, combining our present data with those from previous studies, a model can be proposed for natural deastringency in C-PCNA persimmon. As presented in **Figure 9**, the aldehyde-mediated coagulation effect should be first regarded as a rather important reason for the natural loss of astringency in C-PCNA persimmon. Specifically, pyruvate, one of the key products of glycolysis in flesh and seed, is converted by PDC to acetaldehyde, which then combines with soluble PAs, resulting in the natural loss of astringency in C-PCNA persimmon at the last stage of fruit development. During this process, ADH and aldehyde dehydrogenase-2 (ALDH2) are involved in the metabolic balance of acetaldehyde. In addition, the natural removal of astringency in C-PCNA persimmon occurs via the dilution of tannins as the fruit grows larger.

In summary, based on cDNA-SSAP and RNA-Seq data, six novel *DkPK* genes were isolated using RACE-PCR and genome walking. In C-PCNA persimmon, the expression pattern of *DkPK1* during the last stage of fruit development was positively correlated with a decrease in the soluble PA content. Phylogenetic analysis and subcellular localization further confirmed that DkPK1 is located in the cytoplasm. Furthermore, transient over-expression of *DkPK1* in persimmon leaves led to a significant reduction in soluble PA levels and an increase in the transcript levels of acetaldehyde-related genes *DkPDC1, -3, -4*, and *-5* as well as *DkADH1* and *-3*. Taken together, our results suggest that *DkPK1* may be involved in the natural removal of astringency in C-PCNA persimmon. This is the first report on several novel full-length *DkPK* genes as well as their putative roles in the natural loss of astringency in C-PCNA persimmon. Our study will be helpful for understanding the mechanism of astringency removal and for the breeding of PCNA cultivars in the future.

## Author Contributions

ZL, XD, and CG conceived and designed the experiments; CG carried out the experiments; CG, WC, and RM participated in the data analysis; CG wrote the manuscript; ZL, QZ, and XD helped to draft and review the manuscript. All authors read and approved the final manuscript.

## Conflict of Interest Statement

The authors declare that the research was conducted in the absence of any commercial or financial relationships that could be construed as a potential conflict of interest.
