# Supplementary material for: Isolation and Characterization of DkPK Genes Associated with Natural Deastringency in C-PCNA Persimmon
Source: Front Plant Sci. 2016 Feb 17;7:156. doi: 10.3389/fpls.2016.00156 (PMC4756295; doi:10.3389/fpls.2016.00156)
Supplement: Supplementary file 1 [file Table_1.DOC]

**Table S1. Sequences of the primers used for gene isolation and expression analysis.**

|  | **Gene** | **Primary PCR (5' to 3')** | **Secondary PCR (5' to 3')** |
| --- | --- | --- | --- |
| 3'RACE | *DkPK1* | CTGGAAAGCCTGCTGTGGTGACTC | GACCAACTCGTGCCGAAGCAACTG |
| Full-length | *DkPK1* | GGTGCGAATCTTCAGAGTCCT | AGGCCATAGTTCCCAACACTC |
|  | *DkPK2* | GTAGGGTTGGTGGCGTTGC | AGGCTCCATTCTTATTTAAGTGC |
|  | *DkPK3* | TCTCACTAAATGGCATAAAC | GCTCGCAATTAACCACAAA |
|  | *DkPK4* | AATGACAATCCAACTAGGGAAGT | TTTAGAGGCGGCGATTTCT |
|  | *DkPK5* | TATGTAATTGGATGGCTCAA | CCAAGGCAATGCAAGGAGG |
|  | *DkPK6* | CTTGAATGGCTATCTTGAC | CATGGATAATTTACCTTGTT |
| Real-time  PCR | *DkPK1* | TTCACGTCATCCACCTCCA | AAGGGTGACACCATCTTTGTT |
|  | *DkPK2* | CCGAGGTGATATTGGAAGC | ACCCAGCAAAATGGAGGTT |
|  | *DkPK 3* | GTGGTGGCATGAAAAGCAA | CATCCAACTCCAACGAGGG |
|  | *DkPK4* | AGACATATTCAGACGAGCA | GAGCAAATCCAGTACCTAC |
|  | *DkPK 5* | TCACTGTCATATACTCGGC | ATAATTCCATCTGCTTCCT |
|  | *DkPK6* | ATGCTGATGCCCTCATGTT | ATCCGACTGCTGGTTGTCT |
| Genome walking | *DkPK1* | AGAAGCCTCTTGACCTTGATGGGG | GCTTCGTCGTAGACTTCACGGCGG |
